# Systemic Melioidosis Presenting as Septic Arthritis

**DOI:** 10.7759/cureus.25029

**Published:** 2022-05-15

**Authors:** Vaishnavi Arunpriyandan, Mayurathan Pakkiyaretnam, Maheswaran Umakanth

**Affiliations:** 1 General Medicine, University Medical Unit, Teaching Hospital Batticaloa, Colombo, LKA; 2 Medicine, University Medical Unit, Faculty of Health-Care Sciences, Teaching Hospital Batticaloa, Batticaloa, LKA; 3 Clinical Medicine, Eastern University, Sri Lanka, Colombo, LKA

**Keywords:** emerging disease, eradication of melioidosis, systemic melioidosis, septic arthritis, melioidosis

## Abstract

Melioidosis is a pyogenic infection that is potentially fatal, caused by the bacterium *Burkholderia pseudomallei*, which is commonly a soilborne pathogen.It is endemic in the Indian subcontinent, northern Australia, and Southeast Asia. Melioidosis has a wide spectrum of clinical manifestations that can mimic various diseases. Septic arthritis is a rare but well-known clinical presentation. Here, we report a case of an adult presenting with acute knee joint pain and swelling. He was subsequently found to have septic arthritis with other system involvement and diagnosed as a case of melioidosis; he responded well to the treatment.

## Introduction

*Burkholderia pseudomallei* a gram-negative, aerobic bacillus. It is endemic to the tropical belt countries of the world, including Sri Lanka. It is frequently seen in patients with diabetes, chronic lung disease, or heavy alcohol use [1]. The most common risk factor for melioidosis is occupational exposure to muddy water, commonly in rice-based agriculture. *Burkholderia pseudomallei* can be isolated from water samples taken from rice paddy fields in endemic areas. Due to these factors, Sri Lanka has a favorable environment for this organism. Pneumonia (50%) and solid organ abscess are the common manifestations. Genitourinary infection, skin involvement, and disseminated bacteremia without any evidence of foci are the other presentations [2]. Mortality rates are high, ranging from 14% to 40% in spite of ideal therapy. Recurrence is common unless long courses of targeted therapy are given [1,2].

## Case presentation

A 55-year-old patient with well-controlled diabetes mellitus for seven years presented with intermittent high spiking fever, associated right knee joint pain, and swelling for 10 days duration. The fever was intermittent in nature, which was associated with chills and rigors. He also complained of right hypochondriac pain, which was continuous and dull in nature, and continuous pain without radiation. He had significant myalgia, headache, and arthralgia. He developed severe pain, redness, and swelling with restricted movement of the right knee joint after two days of admission. Occupational history included a recent exposure to working in muddy water. No redness or swelling of any other joints, yellowish discoloration of the eyes, pale stools, or dark urine were noted. He did not complain of any intractable pruritus, lower urinary tract symptoms, or urethral discharge. There was no difficulty in breathing, productive cough, or any alteration in bowel habits.

He had no history of valvular heart disease and exertional dyspnea or any contact history with COVID-19. He denied any recent travel or history of blood transfusion. He was unable to perform his daily activities due to intractable pain. There was no significant medical history or known allergies. The patient was monogamous and denied any sexual promiscuity, smoking, alcohol consumption, or recreational drug use. On examination, he was ill-looking and febrile without conjunctival suffusion or palpable lymph nodes. The right knee joint was swollen with tenderness and warmth. Other system examinations revealed no abnormalities. His blood investigations are presented in Table 1.

**Table 1:** List of investigations with results AST: aspartate aminotransferase; ALT: alanine transaminase; ALP: alkaline phosphatase; PT-INR: international normalized ratio for prothrombin time; UFR: urine full report

Parameter	Value	Normal reference
Full blood count		
WBC	18,000/dL	4,000-11,000/dL
Hemoglobin	9 g/dL	12-16 g/L
Platelets	356,000/dL	150,000-350,000/dL
CRP	259 mg/dL	0-6 mg/dL
ESR	53 mm/first hour	0-29 mm/hour
UFR	No abnormality	
Blood picture	Normocytic normochromic anemia with features of ongoing sepsis	
AST	97 U/L	10-35 U/L
ALT	100 U/L	10-40 U/L
ALP	249 U/L	41-133 U/L
Total protein	64 g/L	64-82 g/L
Serum albumin	24 g/L	34-50 g/L
Serum globulin	49 g/L	22-48 g/L
PT-INR	1.1	0.9-1
Total bilirubin	51.5 micromol/L	2-21 micromol/L
Direct bilirubin	39.4 micromol/L	0-8 micromol/L
Indirect bilirubin	12.1 micromol/L	0-12 micromol/L
GGT	335 u/L	15-85 u/L
Joint fluid aspiration		
Leucocytes	>50,000	
Gram stain	Negative	
Culture	Negative	
Ultrasound of the knee joint	Fluid collection noted within the joint space with thick material within it, more suggestive of septic arthritis	
Abdominal ultrasound	Hypoechoic solitary lesion measuring 4.6 × 5.7 cm in size in the subdiaphragmatic region of the right lobe of the liver (segment vii) favoring an early liver abscess	

**Figure 1:**
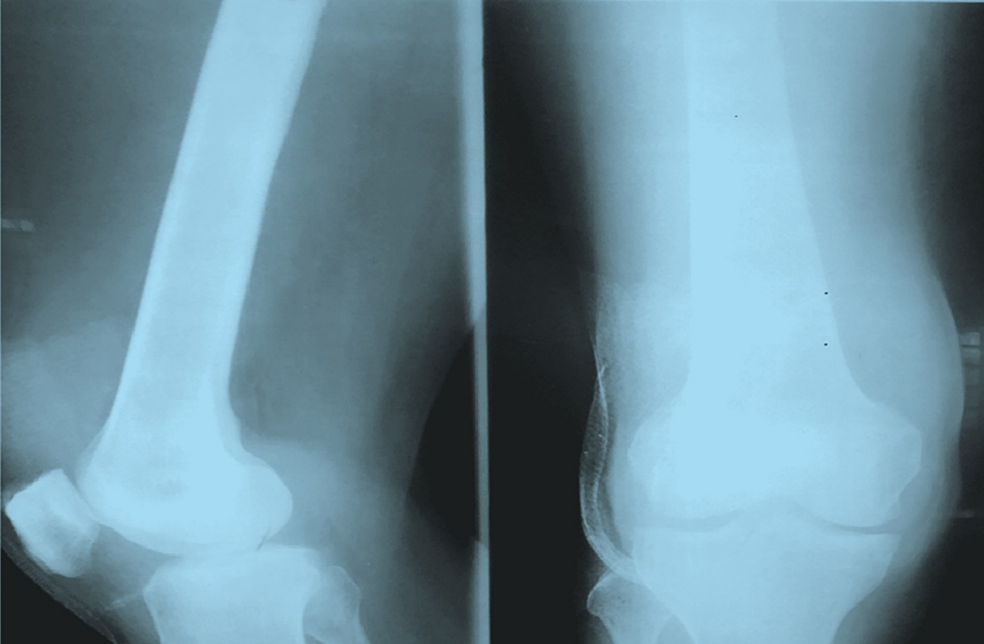
X-ray of the right knee joint showing soft tissue swelling

**Figure 2:**
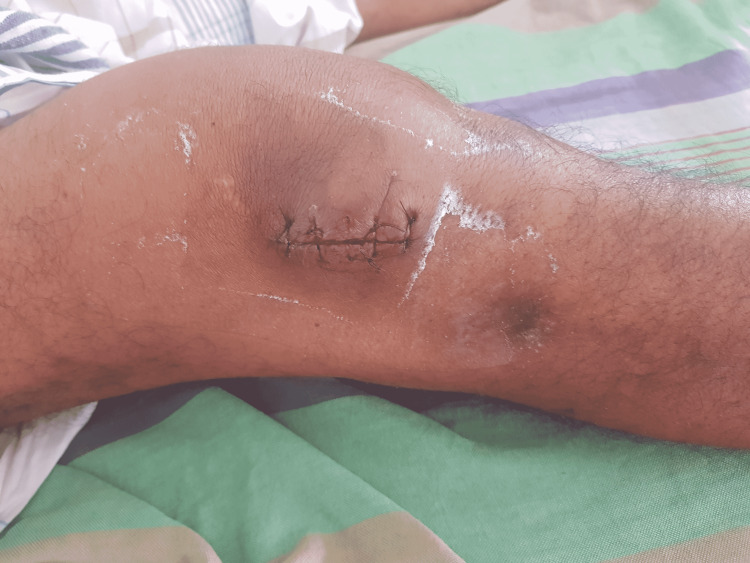
Right knee joint sutured following arthrotomy and drainage tube kept in situ for 72 hours

Following the aspiration of right knee joint fluid and imaging (Figure 1), he was managed as septic arthritis with empirical antibiotics and arthrotomy for pus drainage (Figure 2).

**Figure 3:**
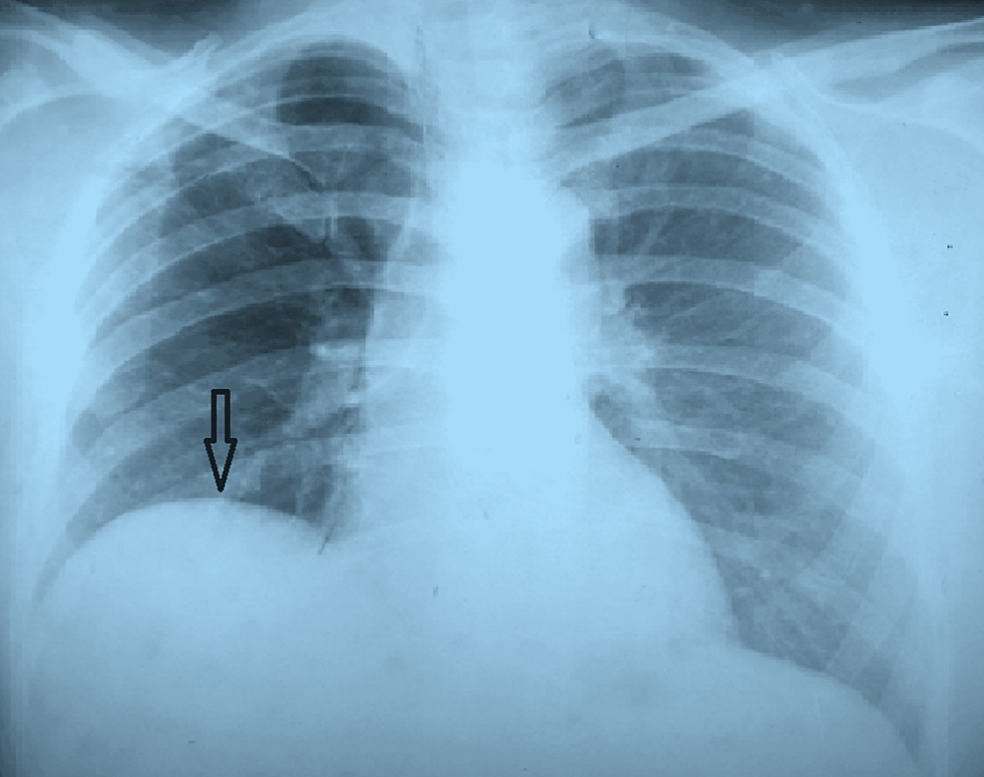
Chest X-ray showing right diaphragmatic elevation due to liver abscess There was only mild hepatomegaly on abdominal examination as the liver has expanded upward, which can be clearly seen in the chest X-ray.

**Figure 4:**
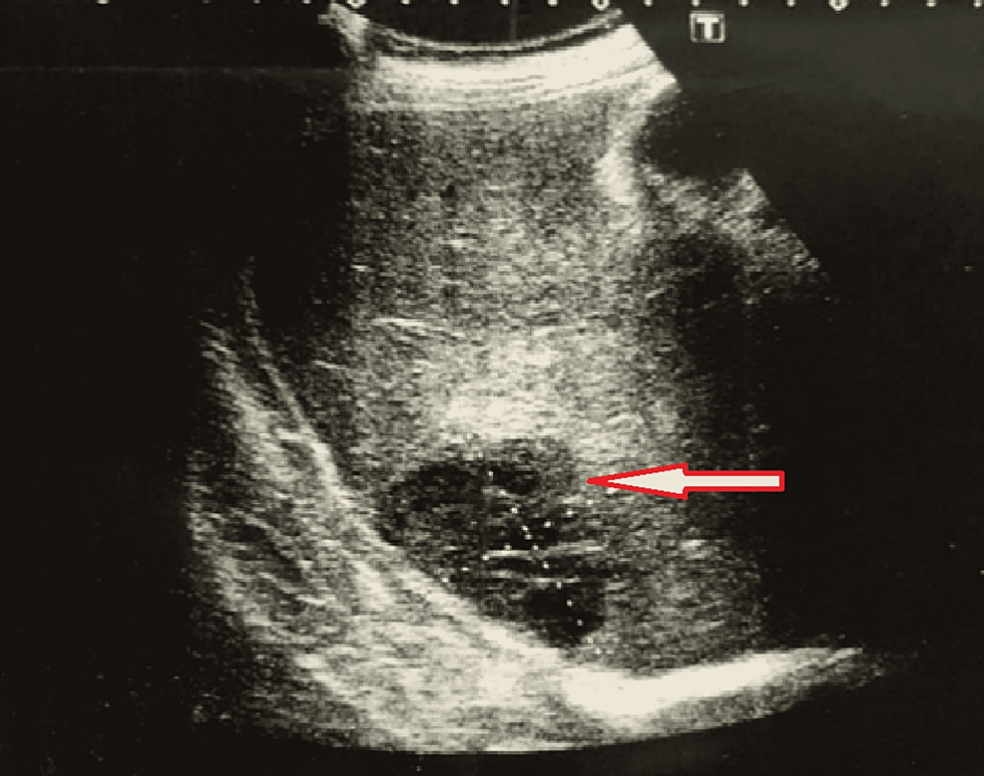
Ultrasound of the abdomen revealing hepatic abscess Arrow indicates a hypoechoic solitary lesion measuring 4.6 × 5.7 cm in size in the subdiaphragmatic region of the right lobe of the liver (segment vii) favoring liver abscess.

On the second day of admission, the patient developed jaundice and was also found to have mild hepatomegaly. Blood workup showed a doubling of initially normal serum bilirubin and an increased direct fraction of bilirubin. Urgent ultrasound of the abdomen revealed a hepatic abscess (Figures 3, 4).

The patient was started on intravenous ceftazidime under high index suspicion of melioidosis since the definitive investigations require a longer time to confirm the diagnosis. The antibiotic regime was continued for 14 days. Later, the serum antibody and blood culture both became positive for melioidosis, and the patient also improved with targeted therapy. We discharged him on oral cotrimoxazole for three months and noted complete recovery at his subsequent follow-up.

## Discussion

Melioidosis is an emerging infectious disease [3]. Although it is endemic in certain countries such as Thailand, Australia, and the Indian subcontinent, there are reports of new cases in regions with no prior exposure to melioidosis [1]. Percutaneous inoculation is the major mode of spread, especially in people who are in contact with soil and water on a regular basis [1,4]. Other less common forms are inhalation or intake of contaminated food and water. Melioidosis is well known for its seasonal variation, and the majority of infections occur during the rainy season [1]. The incubation period is 1-21 days, while some cases demonstrated a prolonged period of latency of up to 62 years [1,2]. *Burkholderia pseudomallei* enter macrophages and reproduce for prolonged periods of time. Although phagocytes try to kill the bacteria, they can escape endocytic vacuoles and move into the cytoplasmic space. They can also infect other cells via actin-based membrane protrusions [1].

Melioidosis has an extended spectrum of clinical signs and symptoms. It can present as an acute fulminant disease to chronic indolent infection mimicking tuberculosis or malignancy. An extensive study carried out in the tropical regions of Australia for two decades identifies pneumonia as the common presenting feature, which was found in 50% of cases, followed by genitourinary infection, skin infection, and bacteremia without evident focus [2,4]. Only 4% of cases present with septic arthritis, which was the principal presentation of our patient. Approximately one-fifth develop septic shock, and overall, 50% have bacteremia on initial presentation. Most of the patients infected with melioidosis are found to have risk factors including diabetes, significant alcohol use, chronic lung disease, chronic kidney disease, and thalassemia. However, steroid therapy and malignancy were only associated in less than 5% of cases [1,2,5]. Our patient also had diabetes but for a short duration, which was well controlled with a single oral antidiabetic agent.

The gold standard for diagnosis is a positive culture for *B. pseudomallei* from any clinical sample, which in our patient's case was a positive blood culture. We would have encountered diagnostic difficulties if the culture had been negative due to the nonavailability of serological or genetic-based testing at the hospital where the patient was treated. The diagnosis of acute or chronic melioidosis remains challenging because strong laboratory facilities with bacteriology are necessary [6]. Due to its high fatality rate, in a setting where there is a delay in obtaining culture investigations, the prompt treatment of melioidosis could be crucial prior to making a definitive diagnosis.

Due to the refractory nature of the melioidosis infection, a prolonged course of appropriate antibiotics should be given. An initial intensive phase should include intravenous ceftazidime or meropenem for at least 10-14 days, followed by oral eradication therapy, which comprises trimethoprim-sulfamethoxazole (TMP-SMX) taken for 3-6 months, with or without doxycycline [6]. One recent study demonstrated that TMP-SMX monotherapy is not inferior to TMP-SMX with doxycycline. In the presence of contraindications, amoxicillin-clavulanate can be used as an alternative agent for eradication therapy [7].

Mortality rates in melioidosis vary from 14% to 40% [2]. Recurrence occurs in one in 16 patients, usually in the first year following the initial disease presentation. Among them, around 75% are due to relapses from persistent infection, with the remainder due to reinfection [8].

Blood culture positivity is not commonly seen in most of the reported cases of melioidosis with an atypical presentation such as arthritis. Although the patient had diabetes, it was well controlled with a small dose of oral metformin, and he did not have any other risk factors. As per our knowledge, a significant immunosuppressed state was commonly seen with disseminated melioidosis.

## Conclusions

This case highlights the need for more widespread awareness of the disease. We would like to stress the importance of suspecting and clinically diagnosing melioidosis in patients with such atypical presentations in endemic areas. Furthermore, due to the high mortality of this disease, the early commencement of treatment for melioidosis without waiting for definitive investigations should be considered.
